# A Data-Driven Loose Contact Diagnosis Method for Smart Meters

**DOI:** 10.3390/s25123682

**Published:** 2025-06-12

**Authors:** Wenpeng Luan, Yajuan Huang, Bochao Zhao, Hanju Cai, Yang Han, Bo Liu

**Affiliations:** School of Electrical and Information Engineering, Tianjin University, Tianjin 300072, China

**Keywords:** smart meters, screw terminal, voltage differentials, loose contact, arc fault, LOF method, MLR method

## Abstract

In smart meters, loose contact at screw terminals can lead to prolonged overheating and arcing, posing significant fire hazards. To mitigate these risks through early fault detection, this study proposes a data-driven framework integrating the Local Outlier Factor (LOF) and Multiple Linear Regression (MLR) algorithms. Voltage differentials, extracted from operational data collected via a simulated multi-meter metering enclosure, are leveraged to diagnose terminal contact degradation. Specifically, LOF identifies arc faults, characterized by abrupt and transient voltage deviations, by detecting outliers in voltage differentials, while MLR quantifies contact resistance through regression analysis, enabling precise loose contact detection, a condition associated with gradual and persistent voltage changes due to increased resistance. Extensive validation demonstrates the framework’s robustness, outperforming conventional centralized methods in diagnostic accuracy and adaptability to diverse load conditions.

## 1. Introduction

China has implemented large-scale deployment of advanced metering infrastructure (AMI) as a cornerstone of intelligent power measurement, with smart meters significantly enhancing the efficiency and convenience of electricity management. However, under the combined effects of manufacturing processes, operational lifespan, environmental conditions, and electrical load characteristics, terminal burnout failures in smart meters frequently occur. These failures not only cause power outages but also pose fire risks, presenting substantial threats to electrical safety and resulting in significant economic losses for power utilities [1]. Data from a power utility’s dismantled meter sorting and disposal laboratory indicates that terminal burnout accounts for over 40% of identified failures among fault cases involving both single-phase and three-phase smart meters (encompassing products from 45 single-phase and 34 three-phase manufacturers). Given this evidence, conducting in-depth research on the failure mechanisms of smart meter terminal burnout and establishing a contact failure early warning system are of critical importance for strengthening electricity safety management and reducing the failure rate of metering equipment.

**Figure 1:**
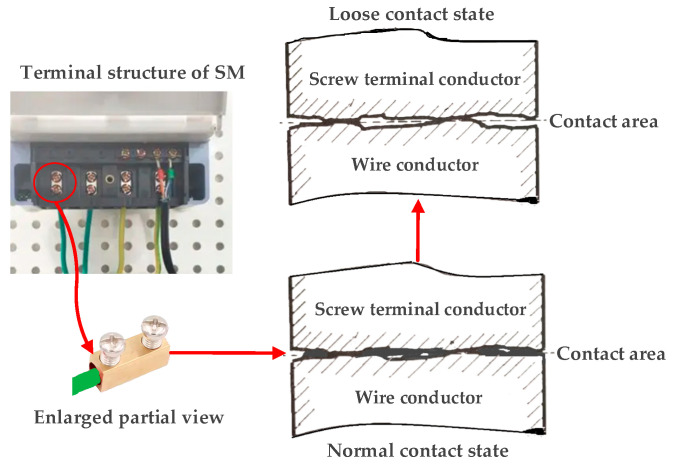
Demonstrative screw terminal to wire contact conditions.

Terminal burnout incidents in smart meters fundamentally originate from current flow through degraded electrical contacts characterized by abnormally high resistance. As illustrated in Figure 1, wires are inserted into the corresponding terminal holes and secured with screws. The surge in resistance stems from progressive reductions in the effective contact area between the terminal and wire [2]. Throughout long-term smart meters operation, compounding factors—including mechanical stress from unreliable connections, thermal cycling induced by power load variations, environmental degradation, and material fatigue—systematically induce terminal loosening, thereby diminishing the conductive interface [3].

Currently, research on detecting loose contacts in electrical wiring remains limited, with existing studies primarily focused on employing supplementary diagnostic equipment for detection. Among these approaches, methodologies such as infrared thermal monitoring employ Stefan–Boltzmann radiation principles to quantify power dissipation in loose connections, achieving validation through comparative voltage drop measurements in industrial furnace environments [4]. Alternative approaches analyze electromagnetic acoustic signatures generated by unstable physical structures at contact interfaces to detect loose connections [5], while computer vision solutions utilize deep-learning-enhanced image comparison against standardized wiring libraries to identify terminal anomalies [6]. Though these methods can effectively detect loose contacts in electrical wires or terminals through non-electrical signal analysis, they rely heavily on extra devices—including thermal cameras, acoustic transducers, or imaging systems—that require physical integration into meter architectures. This intrusiveness incurs prohibitive costs when scaled to China’s operational base of over 300 million widely distributed smart meters [7]. Additionally, ongoing costs for device recalibration and software updates further exacerbate the issue.

It is well known that electrical contact degradation follows a staged resistance trajectory: Initial gradual escalation from tens to hundreds of milliohms during the latent phase, driven by surface oxidation accelerated through localized Joule heating. As oxidative layers accumulate, a self-accelerating failure cycle is triggered, propelling resistance into the hundreds of milliohms to single ohm range, during which, intermittent micro-arcing (glowing connections) transitions to sustained arc faults [8,9]. This progression culminates in abrupt multi-ohm resistance spikes caused by arc-induced contact erosion, accompanied by thermal emissions capable of igniting adjacent combustible materials [4,9]. These resistance variations dynamically modify voltage–current characteristics within the circuit, generating distinct electrical signatures detectable through operational data. Building on these electrical signature dynamics during contact degradation, existing research on electrical contact failure detection has primarily focused on analyzing high-frequency signatures in current waveforms during active arcing. For example, ref. [10] introduced a LightGBM-based feature selection method for arc fault detection using high-frequency current characteristics, while ref. [11] proposed a feature selection strategy combining feature clustering and maximal information coefficient analysis to improve fault detection accuracy under arcing conditions. Advanced methodologies, such as the non-parallel bounded support matrix machine [12], multi-class fuzzy support matrix machine [13], and convolutional-vector fusion network [14], further enhanced arc fault detection capabilities. However, despite demonstrating competence in identifying active arc faults without requiring supplementary detection devices, these methodologies face two inherent constraints: conventional meters cannot acquire high-frequency current signals (mandating costly additional acquisition modules), and detection is feasible only during arcing events with no capability for early warning before arc fault occurrence.

To address the limitations of existing methods for detecting terminal contact faults in smart meters, this study proposes a non-intrusive diagnostic framework utilizing voltage differentials (Diffs) derived from low-frequency operational data of smart meters. By exploiting two intrinsic electrical characteristics—elevated contact resistance during arc faults and protracted incubation behavior of loose contacts—the method enables dual-mode detection of terminal anomalies without supplementary detection devices or high-frequency acquisition modules. This approach transitions fault diagnosis paradigms from reactive post-failure monitoring (during-fault phase) to proactive pre-failure detection (pre-fault phase), thereby establishing early-stage anomaly identification capabilities. The main contributions of this study are listed as follows:

1. Instead of focusing on detecting either loose contacts or arc faults individually, as seen in prior studies, an experimental system incorporating both loose contacts and arc faults is designed to replicate smart meter operation states within metering enclosures. Authentic datasets with validated electrical signatures are constructed using collected signals, establishing a robust foundation for investigating screw terminal failures in smart meters.
2. An interference data cleaning method is developed through analysis of the relationship between voltage fluctuations and load current to eliminate redundant interference. Diffs between co-located smart meters within shared metering enclosures are subsequently established as the primary diagnostic indicator for contact fault identification.
3. A two-stage fault diagnosis method combining Local Outlier Factor (LOF) and Multiple Linear Regression (MLR) is proposed, and its effectiveness is evaluated across two types of load datasets. The stability and generalization of the LOF-MLR method are demonstrated through comparisons with three data-driven methods under the same sample conditions.

The rest of this paper is organized as follows. Section 2 reviews the related work, Section 3 introduces the experiment platform and fault feature extraction, Section 4 presents the LOF-MLR diagnosis methodology, Section 5 validates the LOF-MLR method using diverse datasets, and Section 6 concludes this study.

## 2. Related Work

### 2.1. Evolution Mechanism from Loose Contacts to Arc Faults

Comprehending the degradation progression from terminal loosening to arcing faults and its corresponding electrical signature evolution is crucial for advancing research on terminal contact fault diagnosis. In the loose contact stage, the degradation initiates with progressive contact resistance escalation due to mechanical fretting motion, environmental corrosion, or insufficient torque. Experimental simulations of loose connections under vibration or improper installation reveal oxidation layer formation at contact interfaces, significantly increasing resistance [3,15]. This stage manifests as subtle voltage drops (typically <100 mV) under low-current conditions (even <1 A), with localized heat generation insufficient to trigger arcing. Oxide layer growth creates high-resistance pathways yet remains thermally stable below critical thresholds. Transition to the arc fault stage occurs when sustained resistance escalation induces thermal runaway, elevating temperatures beyond 1200 °C and transforming oxide layers into conductive plasma channels. This process generates glowing connections or micro-arcing, characterized by nonlinear voltage drops ranging from 1 V to tens of volts, accompanied by current concentrations in filamentary paths [16]. Such arcing phenomena exhibit self-sustaining properties, with minimal sustaining currents as low as 0.3 A, and pose fire hazards due to extreme thermal emissions [8]. Crucially, the transition is marked by a shift from quasi-static resistance increases to dynamic, unstable voltage–current relationships, as evidenced by abrupt deviations in operational signatures between these phases [8,16]. Moreover, experimental results confirm that degradation stages exhibit unpredictable durations across both laboratory and field environments [15].

### 2.2. Method Application Context

**Figure 2:**
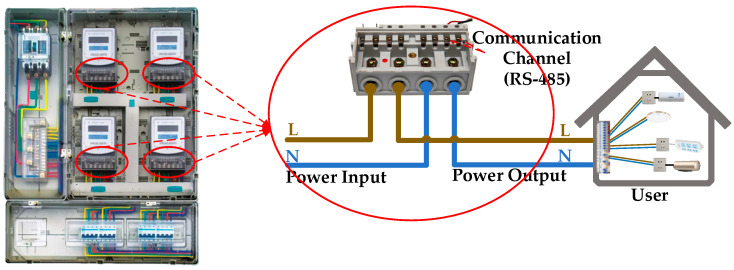
Demonstrative smart meters in the metering enclosure and screw terminal connection.

Before proposing a diagnostic method, it is essential to first analyze the operational context and working mechanisms of smart meters. In China, multiple smart meters are deployed in metering enclosures and powered by a common phase source (single-phase or three-phase supply). Figure 2 shows the smart meters in a typical metering enclosure and their terminals. The ‘Power Input’ connects to the grid via Live (L) and Neutral (N) wires, where the L wire is connected to the phase line (single-phase L or three-phase A/B/C) and the N wire is connected to the neutral line. The ‘Power Output’ connects to the user’s power supply, while the ‘Communication Channel (RS-485)’ receives commands and transmits real-time voltage, current, and communication timestamps from smart meters.

The fundamental principle underlying contact degradation-induced voltage deviations lies in the measurable voltage drop exhibited by smart meters with terminal connection anomalies relative to their nominal supply voltage. Since smart meters within the same enclosure are powered by a common supply source (single-phase or three-phase), those under normal terminal contact conditions exhibit nearly identical voltage profiles closely matching the nominal supply voltage. When terminal loosening or arcing occurs, this equilibrium is disrupted, causing the affected smart meter’s voltage to drop significantly below those of properly connected meters. However, in practical operation, continuous voltage fluctuations caused by grid voltage variations and random user load changes often obscure these fault-induced voltage deviations, rendering standalone voltage monitoring unreliable for fault detection.

### 2.3. Anomaly Detection Techniques

In recent years, existing anomaly detection and diagnosis methods can be generally categorized into two groups, including the physical model-driven method and the data-driven method. However, model-based methods face accuracy limitations in complex power systems due to multivariate interference factors [17], prompting increased adoption of data-driven solutions that utilize operational data for reliable diagnostics.

Data-driven methods have gained prominence for their adaptability, independence from prior assumptions, and strong learning capabilities, showing great potential in efficiently characterizing system operations [18]. A wide range of data-driven anomaly detection methods can be categorized as supervised, semi-supervised, and unsupervised approaches. Supervised methods, such as SVM, decision trees, or neural networks, are typically trained on labeled datasets containing both normal and fault data to detect faults in test samples [19]. However, these methods face significant challenges in acquiring sufficient labeled fault data for training due to the difficulty and cost of data annotation. Alternatively, semi-supervised and unsupervised alternatives demonstrate a high implementation ability by relying on inherent data similarity patterns with minimal labeling requirements [20]. Common methods in this category include correlation analysis [21], Isolation Forest (IF) [22], clustering [23], and the LOF algorithm for identifying potential anomalies. IF requires sufficient samples to build stable tree structures and exhibits unreliable performance on small datasets. Correlation analysis, though intuitive and fast, is highly sensitive to noise interference in the data. Clustering algorithms demand predefined cluster parameters and rely on global distance metrics, incurring high computational costs. Conversely, the LOF algorithm, a density-based method, effectively identifies anomalies by quantifying significant deviations in local density relative to neighboring data points, performing well on low-dimensional, small-sample datasets with dispersed anomalies [19,20].

In reality, multiple fault types often coexist or evolve interactively due to the complexity of the system, exhibiting divergent features. Sole reliance on individual detection methods to identify both fault types often results in increased missed detections and misdiagnoses [24]. Dividing the fault detection problem into specialized sub-models for distinct fault types has proven effective [25]. Compared to serious faults with distinct signatures, early faults are frequently masked by noise and operational variations due to their low magnitude, making them difficult to detect and diagnose. The key to solving these challenges is extracting deep-seated fault features from low signal-to-noise ratio signals. For instance, residual-based methods utilizing the residuals between fault and normal data have been proposed for early fault feature identification in electric hydraulic systems [26], while other research uses MLR and KNN algorithms with three indoor pollutant concentrations to define indoor ventilation states for indoor air quality monitoring and early warning [27]. Therefore, in early fault detection, mathematically modeling the correlation between different parameters can help compensate for the lack of clear early fault features and extend fault diagnosis capabilities. By integrating this approach with data-driven methods for detecting serious faults with distinct signatures, this paper adopts a combination of different anomaly detection techniques for smart meter terminal contact fault diagnosis.

## 3. Data Acquisition and Feature Extraction

This section details how the data are acquired and how features are extracted from them.

### 3.1. Data Acquisition Platform

**Figure 3:**
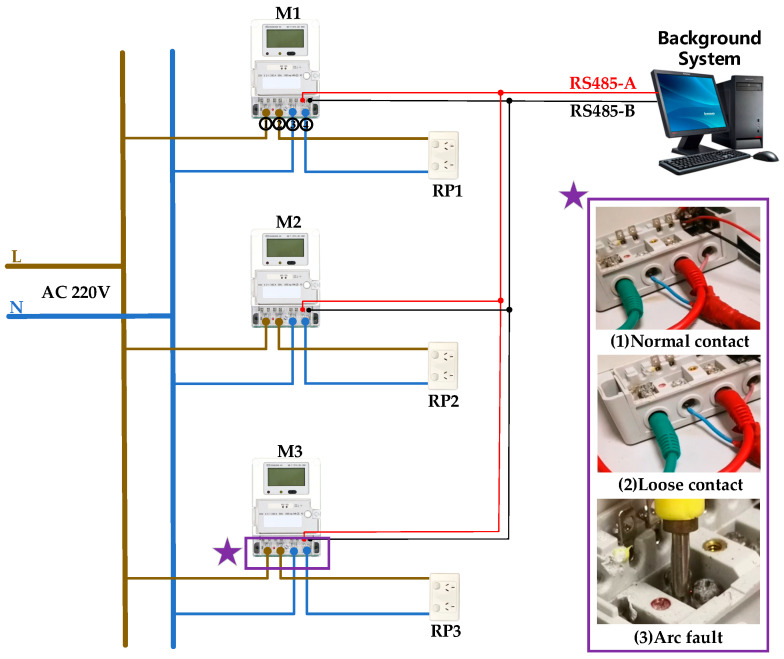
The sketch of the data acquisition platform.

In view of the stochastic nature of data corresponding to terminal looseness and arc faults, this study builds an integrated experimental platform to simulate the terminal connection faults of smart meters, whose wiring configurations replicate those in a real-world shared metering enclosure. The platform is shown in Figure 3.

Employing a point-to-multipoint topology, single-strand copper wires route the L and N lines from the smart grid to multiple smart meters (M1, M2, M3). Standard household receptacles (RP1, RP2, RP3) are connected to the smart meters’ output terminals to allow for flexible load integration based on experimental requirements. The background system, utilizing smart meter-specific communication protocols, simulates the functionality of an AMI system in field-deployed grids, enabling wired communication with smart meters via RS485-A/RS485-B channels in accordance with field standards and automatic recording of operational data (voltage, current, timestamp) from M1–M3.

Under controlled conditions, this platform generates both normal and fault AC signals. The experimental investigation is structured into three phases:

1. Normal contact experiments: M1, M2, and M3 are connected under standardized terminal contact conditions, with voltage and current data systematically recorded across multiple load configurations to establish operational baselines.
2. Loose contact experiments: M3 is designated as the fault terminal while M1 and M2 maintain normal contact. To simulate loose contact conditions, the terminal screw torque on M3 is intentionally reduced, causing partial disengagement between the screw and copper wire. During testing, various loads are applied to all smart meters, with real-time voltage and current monitoring to ensure data accuracy.
3. Arc fault experiments: Arc fault conditions are replicated by inserting the copper wire deep into the terminal hole, making it difficult to visually confirm arcing events. This obstruction prevents accurate dataset labeling. Therefore, the copper wire is repositioned to the upper section of the screw metal block, as depicted in (3) arc fault of Figure 3, to enable controlled validation.

The experimental platform incorporates two load types: resistor loads (static loads) and appliance loads (appliances, mixed static/dynamic loads) to simulate diverse operational scenarios. Resistor loads are designed with fixed current levels (0.4 A, 3.8 A, 7.8 A), combined into distinct portfolios as documented in Table 1. These static loads capture stable voltage characteristics during fault occurrence under controlled conditions, providing a baseline for analyzing fault-specific deviations. Appliances include both dynamic and static components to assess diagnostic robustness under real-world variability. The heat gun operates as a dynamic load, with current continuously varying between 0.25 A and 3.11 A in a sawtooth waveform pattern, characterized by a 1 min cycle and a 2.86 A/min change rate. In contrast, the kettle functions as a static load, maintaining a steady 5 A current during operation. The lamp exhibits minor fluctuations between 0.15 A and 0.35 A, averaging 0.25 A. To replicate the unpredictability of real-world usage, appliance states—activation, deactivation, and intensity variation—are randomized during testing. This approach ensures the evaluation reflects practical scenarios where load changes are irregular and unplanned. Detailed current specifications for all load configurations, including resistor combinations and appliance profiles, are systematically presented in Table 1, enabling reproducibility and transparent benchmarking of the proposed methodology.

In arc fault experiments, instances of continuous strong ignition that cause severe melting of the plastic on wires or terminals are excluded. Each arc fault experiment with resistor loads is conducted for over 0.5 h, and all loose contact experiments last more than 2 h to validate the persistence and reliability of the fault characteristics. Only fully annotated and operationally valid data are retained to ensure usability for subsequent analysis, resulting in a final dataset of 8062 rigorously validated samples. Within this final dataset of 8062 samples, there were 5923 normal connection data points, 1848 loose connection data points, and 291 arc fault data points. The reason for the smaller number of arc fault data points is that arc faults evolve rapidly, leading to shorter experimental durations for this condition.

### 3.2. Experimental Data Analysis

Feature engineering for fault signals involves the application of statistical and mathematical tools, including data preprocessing, analysis, and feature extraction. Key features for detecting smart meter terminal contact faults stem from voltage drops caused by resistance that arises from physical aspects of loose contact or burning arcs.

**Figure 4:**
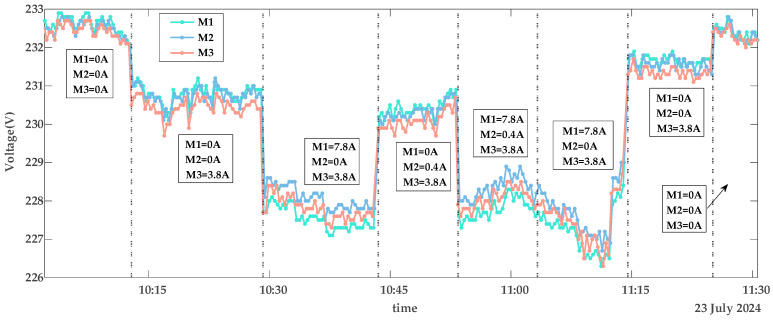
Voltage characteristics of normal contact.

**Figure 5:**
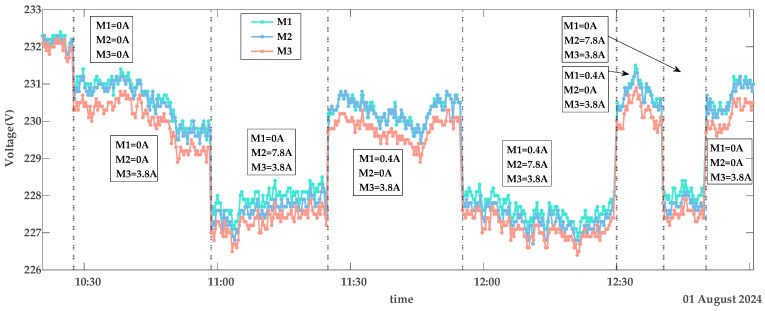
Voltage characteristics of loose contact.

**Figure 6:**
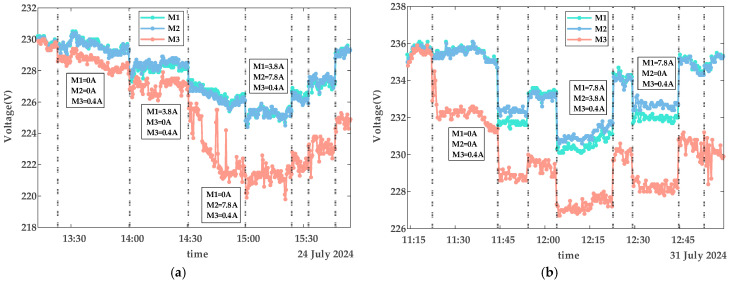
Voltage characteristics of arc faults. (**a**) Description of the scenario where the voltage deviation gradually increases during an arcing fault. (**b**) Description of the scenario where the voltage deviation remains relatively constant during an arcing fault.

The collected data includes smart meter identification codes (IDs), timestamps, voltage, and current values. Using these data, Figure 4, Figure 5 and Figure 6 depict the voltage characteristics of M1, M2, and M3 during normal contact, loose contact, and arc fault stages, respectively. In these figures, the dotted line represents changes in the total current $I_{S}$ within the line. $I_{S}$ denotes the sum of all branch currents, including $I_{1}$, $I_{2}$, and $I_{3}$. Here, $I_{1}$ is the load current on the M1 branch, $I_{2}$ is the load current on the M2 branch, and $I_{3}$ is the load current on the M3 branch. The arrows in Figure 4 and Figure 5 indicate the currents of M1, M2, and M3 within the corresponding $I_{S}$ region. The experimental voltage curves exhibit distinct patterns across contact stages: Under normal conditions, all three voltage curves maintain synchronized trends while inversely correlating with branch load currents—higher loads result in lower voltages, as shown in Figure 4. During loose contact stages, while voltage profiles remain influenced by both total and branch currents, M3’s voltage (red curve) persistently registers the lowest values despite carrying less current than M1/M2 (Figure 5). Arc fault scenarios further amplify deviations in M3’s voltage relative to other branches, with two anomaly patterns observed: progressive deviation escalation over time (Figure 6a) and stable offsets throughout monitoring periods (Figure 6b).

The voltage curves for M1, M2, and M3 exhibit continuous fluctuations depicted in Figure 4, Figure 5 and Figure 6, driven by four key factors: a fluctuation in the power grid (*FPG*); a fluctuation caused by changes in $I_{S}$ (*FTC*); a fluctuation due to variations in branch load currents (*FBC*), as exemplified by voltage curve reordering in Figure 4; and measurement errors of the smart meters (*ME*s), which remain relatively constant per device but differ across meters. As shown in Figure 4, voltage curves diverge among M1, M2, and M3 even under no-load conditions. Among these, *FPG* and *FTC* represent systemic bus-line effects that influence all branch voltages ($U_{1}$, $U_{2}$, $U_{3}$) simultaneously, while *FBC* and *ME*s are localized branch-level fluctuations that affect only the respective branch.

Detailed analysis highlights distinct behavioral patterns: *FPG* dominates voltage variations, with ranges from 226 V to 233 V in Figure 4 and 230 V to 236 V in Figure 6b. *FTC* introduces system-wide deviations, where an 8 A current change in Figure 4 induces 3 V shifts across all branch voltages. Notably, an arc fault-induced fluctuation (*FAF*) specifically affects $U_{3}$, generating about 1 V offsets even under minimal loads (0.4 A), while a voltage fluctuation due to loose contact faults (*FLF*) and *FBC* exhibit comparatively subdued impacts. Collectively, under normal operating conditions, a voltage fluctuation (*VF*) originates from the combined interaction of these factors as modeled in Formula (1):

(1) VF=FPG+FTC+FBC+MEs,

In fault scenarios (loose contact or arc fault), *VF* is cumulatively governed by additional influences defined in Formulas (2) and (3):

(2) VF=FPG+FTC+FLF+FBC+MEs,

(3) VF=FPG+FTC+FAF+FBC+MEs,

The inherent randomness of systemic factors (*FPG* and *FTC*) complicates the isolation of terminal contact faults during diagnostics, particularly for early-stage loose contact detection. To mitigate these systemic interferences, this study employs voltage discrepancy analysis by calculating Diffs between $U_{1}$, $U_{2}$, and $U_{3}$. This method effectively cancels shared *FPG* and *FTC* effects across branches while amplifying fault-specific deviations caused by loose contact or arcing, thereby enabling reliable detection of abnormal deviations.

### 3.3. Feature Extraction

Accurate fault signature extraction requires systematic removal of interference data embedded in raw smart meter data. The Diffs interference stems from smart meter communication polling mechanisms. Within the shared communication channel, the background system sequentially reads M1 ($U_{1}$, $I_{1}$), M2 ($U_{2}$, $I_{2}$), and then M3 ($U_{3}$, $I_{3}$) in cyclic polling intervals, resulting in timestamped data structured such as {[($t_{11}$, $U_{11}$, $I_{11}$), ($t_{21}$, $U_{21}$, $I_{21}$), ($t_{31}$, $U_{31}$, $I_{31}$)], [($t_{12}$, $U_{12}$, $I_{12}$), ($t_{22}$, $U_{22}$, $I_{22}$), ($t_{32}$, $U_{32}$, $I_{32}$)] …, [($t_{1n}$, $U_{1n}$, $I_{1n}$), ($t_{2n}$, $U_{2n}$, $I_{2n}$), ($t_{3n}$, $U_{3n}$, $I_{3n}$)], …}. Critical interference arises when $I_{S}$ changes between $U_{1n}$ and $U_{2n}$ or $U_{2n}$ and $U_{3n}$ readings within the nth polling cycle, generating large Diffs that mimic arc fault signatures. However, $I_{S}$ variation occurring before $U_{1n}$ or after $U_{3n}$ in the nth polling cycle does not distort Diffs analysis. To isolate interference data impacting fault signatures, this study proposes a data cleaning method based on temporal differencing analysis. Given the unknown timing of $I_{S}$ changes, this study defines the following parameters:

(4) $${∆I}_{sn}=\left|{(I}_{1n}+I_{2n}+I_{3n})-(I_{1(n+1)}+I_{2(n+1)}+I_{3(n+1)})\right|,$$

(5) $$D1\left(ni\right)=\left|U_{in}-U_{i(n-1)}\right|,$$

(6) $$D2\left(ni\right)=\left|U_{i(n+1)}-U_{i(n+2)}\right|,$$

**Figure 7:**
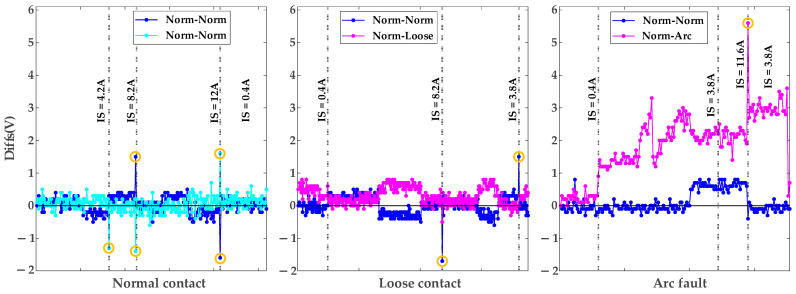
Diffs in normal contacts, loose contacts, and arc faults.

When $I_{S}$ changes (${∆I}_{sn}$) exceed 3 A, the algorithm checks $D1\left(ni\right)$. If $D1\left(ni\right)$ is greater than 1 V, the nth polling cycle data is discarded; otherwise, check if $D2\left(ni\right)$ is greater than 1 V. If it is greater than 1 V, delete the data of the (n+1)th polling cycle. If none of the above conditions are met, the system proceeds to the next polling cycle for data checking and cleaning. Boundary cases (n=1 or n=N−1, where N denotes the final count of polling cycles) are handled by replacing out-of-range data with adjacent cycles. As shown in Figure 7, the data points within the yellow circle represent voltage variations exceeding 1 V caused by load changes. Failure to clean these interference data could result in their misclassification as arc fault signatures. The data-cleaning methodology described above effectively mitigates interference effects induced by load fluctuations, ensuring reliable fault diagnosis.

Threshold selection is empirically validated through experimental and referenced data. The 3 A current threshold is derived from resistor and appliance load tests, where changes in $I_{S}$ below 2 A induce negligible voltage variations in $U_{1}$, $U_{2}$, $U_{3}$, while a 4 A change in $I_{S}$ results in approximately 2 V drops. The 1 V threshold is established based on arc fault experiments showing Diffs exceeding 1 V and supported by transformer data from [28] indicating 0.72 V to 1.22 V drops for 10 (20 or 6) kV/0.4 kV transformers under a full load.

Following interference data cleansing, this study analyzes the distribution ranges of Diffs values under normal contact, loose contact, and arc fault conditions via pairwise voltage subtraction among smart meters within the metering enclosure. Diffs between normal contact and arc fault meters (Norm-Arc) are primarily distributed above 1 V, significantly exceeding those between normal contact meters (Norm-Norm). Comparatively, Diffs between normal contact and loose contact meters (Norm-Loose) exhibit higher concentrations above 0 V relative to Diffs between Norm-Norm, yet the magnitude distinction between these conditions remains subtle, posing significant challenges in distinguishing early-stage loose contact from normal operations.

**Figure 8:**
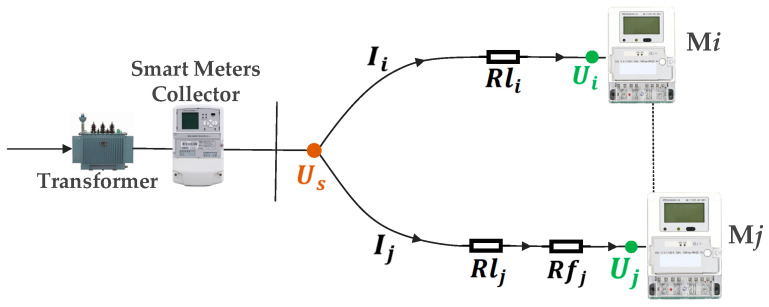
Voltage flow diagram of the metering enclosure.

To further analyze the impact of contact faults on smart meter voltage, this study presents a voltage flow diagram within the metering enclosure [29], revealing systematic deviations caused by terminal degradation. As shown in Figure 8, the analysis assumes that the Mj meter exhibits terminal contact degradation.

Under the normal contact stage, the $U_{i}$ of the Mi is:

(7) $$U_{i}=U_{S}-I_{i}\times {Rl}_{i}-{U\varepsilon }_{i}$$

Under loose contact or arc fault stages, the $U_{j}$ of the Mj is:

(8) $$U_{j}=U_{S}-I_{j}\times {Rl}_{j}-I_{j}\times {Rf}_{j}-{U\varepsilon }_{j},$$

By subtracting $U_{i}$ from $U_{j}$, $U_{i-j}$ can be obtained:

(9) $$U_{i-j}=U_{i}-U_{j}=-I_{i}\times {Rl}_{i}+I_{j}\times ({Rl}_{j}+{Rf}_{j})+({U\varepsilon }_{j}-{U\varepsilon }_{i}),$$

(10) $$U_{i-j}=\beta_{0}+\beta_{i}\times (-I_{i})+\beta_{j}\times I_{j},$$

In (10), $\beta_{0}={U\varepsilon }_{j}-{U\varepsilon }_{i}$, $\beta_{i}={Rl}_{i}$, $\beta_{j}={Rl}_{j}+{Rf}_{j}$. In (7)–(10), i,j=1,2,3, i≠j, which represents the smart meter in the metering enclosure. ${Rl}_{i}$ and ${Rl}_{j}$ denote the combined resistance of circuit wiring and normal terminal contact, while ${Rf}_{j}$ is the resistance introduced by terminal contact failure. ${U\varepsilon }_{i}$ and ${U\varepsilon }_{j}$ are the *ME*s of the Mi and Mj; although not strictly necessary, their inclusion could improve the $\beta_{i}$ and $\beta_{j}$ values by accounting for *ME*s [29], derivable from Diffs under no-load conditions. Known variables $U_{i}$, $U_{j}$, $I_{i}$, and $I_{j}$ are directly obtained from collected operational data. Using the MLR method, $\beta_{i}$ and $\beta_{j}$ are calculated to further diagnose the contact state of smart meter terminals.

### 3.4. Comparison with Other Feature Extraction Methods

To compare the effectiveness of Diffs features, other methods for feature extraction using voltage and current data are calculated for comparison. Specifically, the Dynamic Time Warping (DTW) distance between meter pairs is derived to quantify the similarity features of their operational curves within the metering enclosure. Additionally, statistical analysis of voltage differences is performed to extract skewness features (SKF), and voltage-to-current difference ratios (∆*V*/∆*I*) are analyzed to understand contact resistance distribution patterns across meters.

Analysis of the DTW distance feature under Norm-Norm, Norm-Loose, and Norm-Arc conditions reveals that DTW values are close for Norm-Norm and Norm-Loose but significantly differ from those under Norm-Arc. However, this distinction lacks robustness, as DTW values vary considerably across different datasets, potentially due to voltage and load changes. For the SKF feature, Norm-Norm values typically range from −0.86 to +0.43, Norm-Loose values from +1.94 to +2.93, and Norm-Arc values from −0.45 to +1.36. Beyond exhibiting overlaps, this distribution also shows significant variations in SKF values across different datasets under Norm-Arc conditions (e.g., ranging from −0.13 to −0.45 and +1.16 to +1.36), indicating limited robustness. Similar instability is observed in the Δ*V*/Δ*I* feature, with substantial overlap between normal and fault state distributions and non-robust performance across datasets.

As shown in the voltage curves of Figure 4, Figure 5 and Figure 6, the clear diagnostic signature of terminal contact faults is a voltage drop caused by increased contact resistance. However, excessive feature computation steps tend to dilute this signal. This fundamental limitation underscores the rationale for selecting Diffs as the primary fault indicator.

## 4. Contact Fault Diagnosis Method

**Figure 9:**
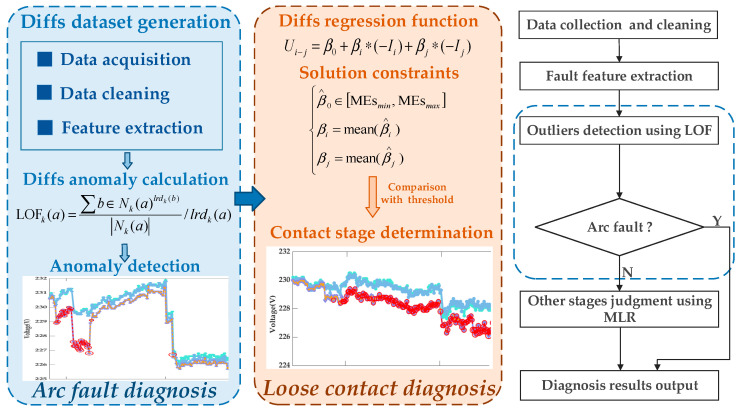
Terminal contact fault diagnosis framework.

This section presents the LOF-MLR framework for contact fault diagnosis, integrating the LOF and MLR methods. The selection of this combined approach is driven by the distinct characteristics of the fault types and dataset: arc faults exhibit significant variability in Diffs signatures, making them prominent yet transient anomalies detectable via LOF’s density-based outlier analysis. Our dataset—comprising low-dimensional voltage, current, and timestamp features with moderate scale—aligns with LOF’s strength in localized anomaly detection. However, the subtle voltage deviations caused by loose contacts result in overlapping Diffs between normal and loose contact conditions, rendering LOF insufficient for standalone diagnosis. To address this, MLR is integrated to quantify the relationship between voltage differentials and currents (as derived in Section 3), enabling precise resistance-based detection of gradual loose contact degradation. As illustrated in Figure 9, the framework operates on the Diffs dataset generated through data acquisition, cleansing, and fault feature extraction processes described in Section 3 and comprises two principal parts as follows. In these two parts, the curves in different colors represent the voltages of smart meters within the metering enclosure.

1. Arc fault diagnosis: The LOF algorithm performs outlier detection on the Diffs dataset. For smart meter IDs exhibiting continuous outliers, the corresponding points on their voltage curves are marked as arc faults. Due to the irreversible nature of arc faults, the remaining non-arc points in the sequence are labeled as loose contacts.
2. Loose contact diagnosis: Based on the linear relationship between voltage drops, current, and contact resistance established in Formula (10), the MLR model calculates contact resistance for datasets devoid of arc faults. Loose contact diagnosis is achieved through comparative analysis against the predefined threshold.

The diagnostic efficacy of the LOF-MLR framework stems from the integrated application of its two core methodologies. The LOF algorithm detects arc fault signatures via density-based anomaly detection in Diffs sequences, whereas the MLR model evaluates contact degradation by resolving multivariate relationships among operational parameters. These complementary techniques are further detailed in subsequent sections, which clarify their theoretical foundations and implementation mechanisms to fully explain the diagnostic process.

### 4.1. Outlier Detection Using LOF

LOF is a density-based method designed for outlier detection. By comparing the local density of a sample to that of its neighbors, it quantifies the sample’s abnormality as a numerical score [20]. For a data point $a(U_{a},I_{a})\in D_{M}$ in Diffs datasets and its kth nearest neighbor $b(U_{k},I_{k})$ from $D_{M}$, the distance between these two points can be calculated as:

(11) $$D_{k}\left(a\right)=\sqrt{{\left(U_{a}-U_{k}\right)}^{2}+{\left(I_{a}-I_{k}\right)}^{2}},$$

Here, the Euclidean distance calculates the spatial distance between points, while other metrics, such as Manhattan distance, Mahala Nobis distance, or Chebyshev distance can also be utilized for this purpose.

Then, the implementation of LOF can be further summarized as follows:

Step 1: Given *k*, the *k*-distance neighborhood of $a(U_{a},I_{a})$ is defined as the set of all objects $b(U_{b},I_{b})$ whose distance from $a(U_{a},I_{a})$ does not exceed the *k*-distance $D_{k}\left(a\right)$, which can be presented as:

(12) $$N_{k}\left(a\right)=\left\{b\in D_{M}|D\left(a,b\right)\leq D_{k}\left(a\right)\right\},$$

Step 2: Then, the *k*th reachability distance from $b(U_{k},I_{k})$ to $a(U_{a},I_{a})$ can be described as:

(13) $$reach-{dist}_{k}(a,b)=max\left\{D_{k}\left(b\right),D\left(a,b\right)\right\},$$

Step 3: Subsequently, the local reachability density of $a(U_{a},I_{a})$ can be given by:

(14) $${lrd}_{k}(a)=\frac{\left|N_{k}\left(a\right)\right|}{{\sum b\in N_{k}\left(a\right)}^{reach-{dist}_{k}(a,b)}},$$

Step 4: Finally, the outlier score of $a(U_{a},I_{a})$ can be determined by:

(15) $${LOF}_{k}(a)=\frac{{\sum b\in N_{k}\left(a\right)}^{{lrd}_{k}\left(b\right)}}{\left|N_{k}\left(a\right)\right|}/{lrd}_{k}(a),$$

Point $a(U_{a},I_{a})$ is considered an outlier if the value of ${LOF}_{k}(a)$ exceeds the specified threshold $T_{h}$.

The three parameters of LOF—k, $T_{h}$, and the method for calculating $D_{k}\left(a\right)$—directly influence outlier detection results. However, unsupervised optimization of these parameters remains challenging due to the varying data distribution patterns. To address this, this study develops a fault-free LOF model trained exclusively on Diffs from normally connected smart meters, whose robustness depends on two key factors: training dataset scale and representativeness. Input parameters specify the data type (Norm-Norm Diffs) and scale, with the training set encompassing four distinct current levels (0.4 A, 3.8 A, 7.8 A, 11.6 A). To balance computational efficiency and data diversity, one-quarter of observations from each current level are selected for training, enabling the model to output identified outliers in test datasets.

Furthermore, given the overlapping Diffs features between loose and normal contact stages—a limitation of LOF’s density-based detection—this study incorporates Formula (10) (derived in Section 3.3) into the contact fault diagnosis framework. To operationalize this formulation, the MLR method is employed to solve the coefficients of Formula (10), thereby obtaining quantitative contact resistance values.

### 4.2. Solution of Resistance Using MLR

A linear regression model describes the behavior of the dependent variable y by relating it to the dependent variable x and random noise ε, typically expressed in the following form [27]:

(16) $$y_{t}=X_{t}\times \beta +\varepsilon_{t},$$

where $\varepsilon_{t}$ is a random value term, $X_{t}=(1,x_{1t},x_{2t},x_{3t},\ldots ,x_{kt})$ represents a vector of k explanatory variables, $\beta ={\left(\beta_{0},\beta_{1},\beta_{2},\ldots ,\beta_{k}\right)}^{T}$ is the value of coefficients, and $y_{t}$ is the dependent vector at time t for t=1, 2, 3,…,N. In this study, Diffs ${\left(U_{i-j}\right)}_{t}=y_{t}$ is explained by a linear model using the current $x_{1t}\;$($-I_{it}$) and current $x_{2t}\;(I_{jt})$, where i and j represent different smart meters within a metering enclosure, and t is the operation time of the smart meters in the enclosure. Thus, the above equation can be rewritten as:

(17) $$y_{t}={\left(U_{i-j}\right)}_{t}=\left(-I_{it}\right)\times \beta_{1}+I_{jt}\times \beta_{2}+\beta_{0}+\varepsilon_{t},$$

The parameters $\beta_{1}$ and $\beta_{2}$ are determined using collected data from Section 3, estimated via the least squares method to minimize the squared sum of error variables. Vector $\beta ={\left(\beta_{0},\beta_{i},\beta_{j}\right)}^{T}$ represents the values that minimize this squared sum:

(18) $$min\sum_{t=1}^{N}\varepsilon_{t}^{2},$$

During solving, $\beta_{0}$ is constrained to the range of *ME* values for the solution, and the following must be satisfied:

(19) $$\beta_{0}\in [{MEs}_{min},{MEs}_{max}],$$

The problem can be expressed in matrix form as follows:

(20) $$min\;{\varepsilon_{t}}^{T}\varepsilon_{t}\;s.t\;\varepsilon_{t}=X_{t}\beta -y_{t},$$

By substituting ε into the objective function, we obtain an unconstrained optimization problem:

(21) $$\min{\left(X_{t}\beta -y_{t}\right)}^{T}(X_{t}\beta -y_{t}),$$

Taking the derivative and setting it equal to zero results in the solution being:

(22) $$2{X_{t}}^{T}X_{t}\beta -2{X_{t}}^{T}y_{t}=0\to {{\beta =(X_{t}}^{T}X_{t})}^{-1}{X_{t}}^{T}y_{t},$$

where $X_{t}={\left(X_{1},X_{2},X_{3},\ldots ,X_{N}\right)}^{T},$ $\beta ={\left(\beta_{0},\beta_{i},\beta_{j}\right)}^{T}$, and $y={\left(y_{1},y_{2},y_{3},\ldots ,y_{N}\right)}^{T}.$ The $\beta_{i}$ and $\beta_{j}$ are derive from solving with Diffs, each consisting of (n−1) values, with their final value calculated as:

(23) $$\begin{array}{c} \;\;\;\;\;\;\;\;\;\;\bar{\beta_{i}}=mean\left(\beta_{i}\right)\\ \bar{\beta_{j}}=mean\left(\beta_{j}\right), \end{array}$$

The parameters $\bar{\beta_{i}}$ and $\bar{\beta_{j}}$, derived from the MLR model, are fixed values representing the line segments of smart meters Mi and Mj within the metering enclosure. These values serve to determine whether there is a faulty terminal contact over the entire time series, providing a holistic assessment rather than evaluating individual data points. In practical fault scenarios, loose contacts or arc faults at Mj cause $\beta_{j}$ to exhibit a slight increase compared to $\beta_{i}$. However, this trend alone cannot reliably determine terminal contact stages. Prior experiments under varying torque levels demonstrate that contact resistances exceeding 80 mΩ pose safety risks, necessitating replacement before reaching 250 mΩ [15]. While the wire specifications in this study align with those in [15] except for copper wire cross-sectional area differences, the contact resistance thresholds remain applicable. Furthermore, empirical analysis of voltage drops distributions across 0.4–11.6 A reveals that loose contact faults predominantly induce voltage drops between 0.1 V and 1 V. Based on these findings, a 100 mΩ threshold is established for identifying loose contacts under standard smart meter wiring configurations. It should be emphasized that loose contact degradation is a gradual process, and the 100 mΩ threshold represents an empirical approximation under existing experimental conditions.

While the constant threshold provides empirical guidance for gradual loose contact degradation, this static approach proves inadequate for arc fault scenarios with dynamically evolving contact resistance. As shown in Figure 6a, arc faults manifest as persistent voltage deviations over time, reflecting continuously escalating contact resistance, whereas loose contacts exhibit quasi-static resistance changes. These temporal feature differences render unified detection criteria ineffective: the LOF algorithm excels at capturing transient arc patterns through dynamic voltage anomaly detection, while the MLR model quantifies steady-state resistance shifts. Neither methodology alone suffices for comprehensive diagnosis due to their differing fault representations. Hence, this study uses the combination of LOF and MLR methods for terminal contact fault diagnosis.

### 4.3. Evaluation Metrics

The proposed LOF-MLR framework is verified by different experiments. To evaluate the LOF model’s diagnostic performance, metrics such as *PPV*, *TPR*, *FAR*, and *F1* are employed [30], derived from a confusion matrix comparing actual and diagnosed results. These metrics are defined as follows: True Positive (*TP*) refers to when the actual class Y is correctly identified as class Y. False Positive (*FP*) refers to when the actual class $\bar{Y}$ is mistakenly classified as class Y. False Negative (*FN*) refers to when the actual class Y is incorrectly labeled as class $\bar{Y}$. True Negative (*TN*) refers to when the actual class $\bar{Y}$ is accurately identified as class $\bar{Y}$. Based on these definitions, the following can be obtained.

Furthermore, as the MLR method evaluates terminal contact conditions via resistance thresholds over operational periods, a supplementary metric—the diagnostic error count—is introduced alongside conventional performance indicators (*PPV*, *TPR*, *FAR*, *F1*). Experimental validation involves eight resistor load datasets and four appliance load datasets, with each dataset containing data from three smart meters, yielding 36 sets of terminal contact state data for diagnosis. These 36 diagnostic units comprised 28 sets of normal contact data, 4 sets of loose contact data, and 4 sets of arc fault data. The error count quantifies misclassified cases within these 36 diagnostic units.

## 5. Frame Results and Discussion

To validate the proposed LOF-MLR method, multiple experiments are conducted using datasets with different fault types, including data from both resistor and appliance load conditions. It is noteworthy that only the normal contact dataset is used for training the LOF model. Subsequently, a comparative analysis is performed using existing fault diagnosis methods, such as *K*-means, DBSCAN, and SVM models. During parameter configuration for these comparative methods, the contact fault categories are defined as two classes (loose contact and arc fault), and parameters are selected to achieve the global optimum for maximizing performance. To ensure a fair comparison, the validation datasets are identical to those used for the proposed LOF-MLR method. The test results of these methods are systematically analyzed and discussed.

### 5.1. Model Validation Using Resistor Load Data

During the progression of terminal contact faults in smart meters, once a loose contact or arc fault occurs and remains unrepaired, the terminal cannot automatically revert to a normal contact state. These faults may alternate between loose contact and arc fault stages over time.

**Figure 10:**
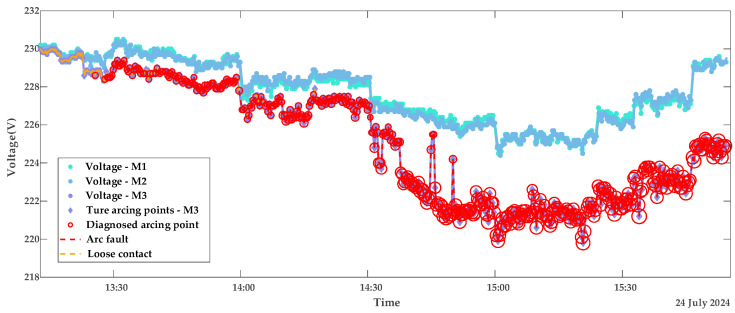
Diagnostic results of contact stages with resistor loads portfolio 1.

**Figure 11:**
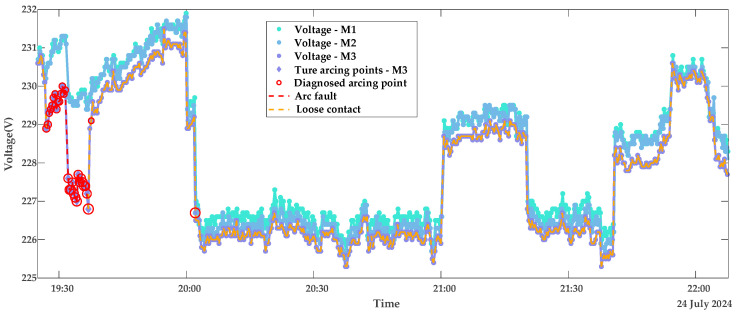
Diagnostic results of contact stages with resistor loads portfolio 2.

**Figure 12:**
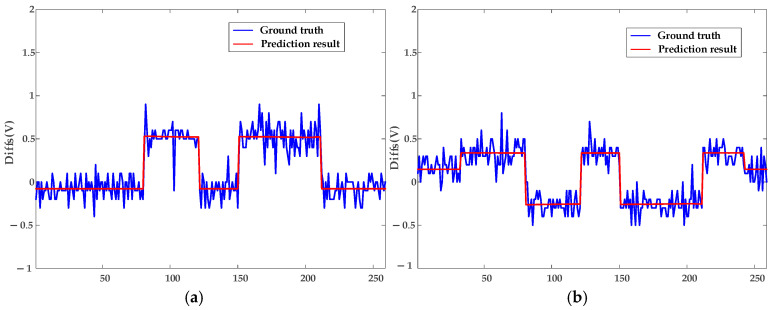
Fitting results of the MLR method for Diffs under resistor loads. (**a**) Description of Norm-Loose Diffs varying under different currents. (**b**) Description of Norm-Norm Diffs varying under different currents.

In this subsection, the model is validated using datasets of loose contacts, arc faults, and mixed faults. The diagnostic results are shown in Figure 10 and Figure 11: The red dashed line indicates the segment with continuous arcing, and the red circles represent the diagnosed outliers, i.e., voltage points with arc faults. The circle sizes reflect the severity of deviation from normal voltages. Figure 12 presents the true and predicted Diffs values from the MLR-based diagnosis on datasets without arc faults, while Table 2 lists the resistances obtained from these tests. It can be seen that normal contact resistances remain within tens of milliohms, whereas those under loose contact exceed 100 mΩ. Additionally, for arc faults causing fixed voltage deviations, MLR tests reveal resistances in the hundreds of milliohms. These results are consistent with the theoretical experimental results in [15].

Non-synchronous data collection due to communication polling introduces fluctuations in Figure 12’s ground truth, as Diffs calculations incompletely eliminate *FPG* effects, and the fixed $\beta_{0}$ fails to fully encompass *ME* ranges. The resistor load operates at constant currents, e.g., experiencing abrupt switching from 3.8 A to 7.8 A instead of gradual increases, explaining the pulse-like predicted waveform in Figure 12.

### 5.2. Model Validation Using Appliance Load Data

**Figure 13:**
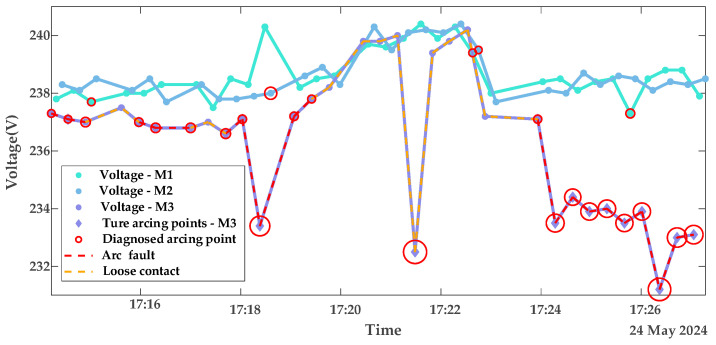
Diagnostic results of contact stages with appliance loads portfolio 1.

**Figure 14:**
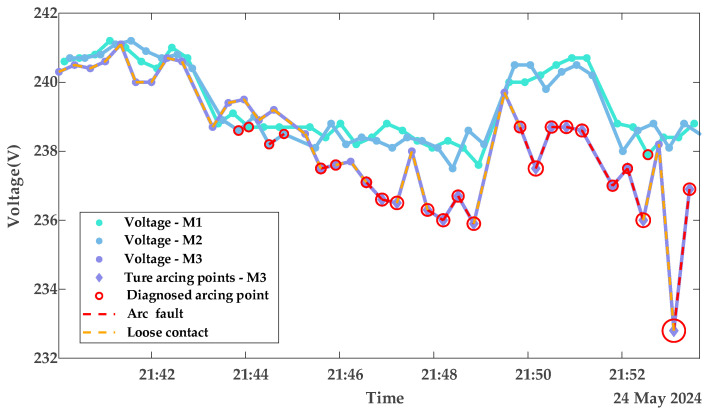
Diagnostic results of contact stages with appliance loads portfolio 2.

**Figure 15:**
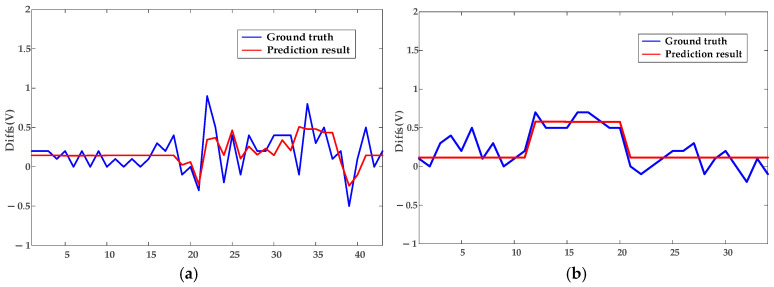
Fitting results of the MLR method for Diffs under appliance loads. (**a**) Description of Norm-Loose Diffs varying under different currents. (**b**) Description of Norm-Norm Diffs varying under different currents.

To further validate the robustness of the proposed model, tests are conducted on datasets collected under appliance loads. Due to the automatic power-off behavior of appliances—for example, the kettle switches off after boiling, with an operating time of approximately five minutes—each appliance load experiment spans about 20 min. Figure 13 and Figure 14 display the diagnostic results for terminal contact faults under appliance load conditions, while Figure 15 shows the true and predicted Diffs values from the MLR-based diagnosis on datasets without arc faults. Tested resistances are summarized in Table 2.

From the diagnostic results in this subsection and the previous subsection, it can be observed that the proposed LOF-MLR method can accurately diagnose the contact fault type on the M3 terminal, both under resistor loads and appliance loads. However, due to the more frequent current variations with appliance loads, the misjudgment rate of normal points under these loads is slightly higher than that under resistor loads. As shown in Figure 13 and Figure 14, besides the detection of arc fault points on the faulty meter M3, fault points were also detected on the voltage curves of the normal meters M1 (green) and M2 (blue). It can be observed that these misjudged points correspond to voltage drops resulting from load changes on the normal meters. Crucially, these misjudgments occur only during current transitions and are non-continuous, thus not affecting overall diagnostic reliability. Table 2 reveals that, alongside the abnormal M3 terminal, the M1 terminal’s contact resistance is marginally higher than that of M2. This discrepancy arises from greater oxidation and corrosion on the M1 terminal compared to the M2 terminal. Additionally, the limited appliance load data results in larger contact resistance variations compared to those obtained under resistor loads.

### 5.3. Ablation Experiments

To verify the necessity of combining LOF and MLR, ablation experiments are conducted using the LOF method alone and the MLR method alone for the detection of both arc faults and loose contacts. Both experiments operate under identical resistor loads and appliance loads with consistent evaluation metrics as the combined method. Performance indicators include *PPV*, *TPR*, and *F1* scores; quantified diagnostic accuracy (higher values are preferred); and *FAR*, which reflects misdiagnosis rates (lower values are preferred).

As shown in Table 3, the experimental results demonstrate that the performance of both the LOF method alone and the MLR method alone is significantly lower than that of the combined experiment under both resistor loads and appliance loads. Specifically, while the LOF method alone shows comparable indicators for arc fault detection to the combined experiment, it fails to detect loose contact faults. This results in two sets of loose contacts being misdiagnosed as normal contacts, leading to a diagnostic error count of 2. This misjudgment pattern is consistently observed under appliance loads as well. For the MLR method alone, an arc fault contact resistance threshold of 300 mΩ is set based on the experimental results from [15]. Because the MLR method evaluates terminal contact conditions via resistance thresholds over the entire operational period, two types of misdiagnoses occur in mixed arc fault and loose contact cases. First, arc faults with a small number of arc points are misjudged as loose contacts. Second, loose contacts are misdiagnosed as arc faults. These misclassifications lead to an increase in the diagnostic error count, a decrease in the number of *TP*, and an increase in the number of *FP* and *FN*. Collectively, these factors result in an overall significant decline in *PPV*, *TPR*, and *FAR* metrics compared to the evaluation indicators of the combined experiment. This issue also shows the same performance under appliance loads. While standalone methods show competence in specific fault types, their inability to handle concurrent or ambiguous fault patterns fundamentally limits diagnostic reliability, thereby proving the essential complementary relationship between LOF and MLR.

### 5.4. Comparison with Deterministic Approaches

To further validate the diagnostic capability of the proposed method, a comparative analysis is conducted against several established techniques. These include *K*-means, DBSCAN, Polynomial Regression (PR), and SVM. For *K*-means and DBSCAN, which are common outlier detection methods, the MLR component is integrated for fairness during comparison with LOF. Additionally, the IF and PR combined method is incorporated into this broader comparison. SVM, as a supervised learning method, serves to compare the overall performance of LOF-MLR, *K*-means-MLR, DBSCAN-MLR, and IF-PR. During this comparison, the number of clusters in *K*-means and DBSCAN is consistently set to 2, and in PR, the order of independent variables is set to the first order to obtain the coefficients for a linear solution. These methods are evaluated under both resistor loads and appliance loads, comprehensively demonstrating and analyzing the advantages and general applicability of the proposed approach.

*PPV*, *TPR*, and *F1* scores quantify diagnostic accuracy, with higher values indicating better performance, while *FAR* reflects the misdiagnosis rate, with lower values being preferable. As shown in Table 3, under resistor loads, LOF-MLR achieves an *FAR* of 0.06 and an *F1* score of 0.98, whereas DBSCAN-MLR attains an *FAR* of 0 and an *F1* score of 0.95, both demonstrating strong arc fault diagnosis. However, under appliance loads, the *FAR* of DBSCAN-MLR is 0.09 and the *F1* score is 0.78, with the diagnostic performance significantly lagging behind LOF-MLR. Notably, *K*-means-MLR performs slightly better under appliance loads than resistor loads—an exception among the tested methods—but requires more parameter tuning and still underperforms compared to LOF-MLR. SVM, which directly classifies Diffs points into normal contact, loose contact, or arc fault stages, exhibits the highest *FAR* due to its sensitivity to transient variations. Compared to the LOF-MLR method, the IF-PR hybrid demonstrates lower diagnostic accuracy, primarily because the IF method tends to misclassify more normal data as arc faults during arc fault point detection. This behavior is consistently observed under both resistor loads and appliance loads.

The last column of Table 3 presents diagnostic error counts across 36 test sets for LOF-MLR, *K*-means-MLR, DBSCAN-MLR, and SVM. LOF-MLR achieves the lowest error count, with a single misclassification attributed to a calculated contact resistance of 105 mΩ, resulting in a normal contact being labeled as loose contact. The results also show that DBSCAN-MLR performs similarly to LOF-MLR under resistor loads but is less effective under appliance loads. The performance of *K*-means-MLR in the diagnostic error count indicator is consistent with its other indicators in Table 3, showing slightly better accuracy under appliance loads compared to resistor loads. SVM exhibits consistent performance across both load types but remains inferior to LOF-MLR. The contact resistance values derived from the PR method are slightly higher than those from MLR, though the overall difference remains within 20 mΩ. This minor discrepancy explains why the PR-based approach yields one additional diagnostic error compared to MLR. Overall, the inferior performance of IF-PR primarily stems from the limitations of the IF component, which introduces systematic misclassification biases. These findings collectively affirm that LOF-MLR offers superior suitability for smart meter terminal contact fault diagnosis, balancing accuracy, robustness, and operational practicality.

### 5.5. Challenges in Application

The LOF-MLR method enables contact state diagnosis of terminal conditions based on smart meters’ operational data, eliminating the need for additional hardware deployment while ensuring reliable diagnostic outcomes. Early detection of loose contact faults provides actionable warnings, which reduces maintenance costs and electrical fire risks. To fulfill the model’s requirement for synchronized voltage and current data from multiple smart meters within the metering enclosure, the diagnostic framework needs to be embedded in smart meter collectors or AMI background systems, enabling self-health diagnostics for terminal contact faults. However, a critical challenge lies in the method’s dependency on low-voltage distribution network topology recognition technology, as diagnostic accuracy is constrained by its reliability in mapping smart meters to their respective metering enclosures.

## 6. Conclusions

This paper proposes the LOF-MLR framework for diagnosing terminal contact faults in smart meters, integrating density-based anomaly detection (LOF) and multivariate regression (MLR). Terminal contact states are classified into three stages—normal contact, loose contact, and arc faults—based on the evolution of terminal faults, with voltage differentials serving as the primary fault indicator. An experimental platform simulating multi-meter operations within a metering enclosure is developed to replicate loose contact and arc faults, generating datasets under resistor and appliance loads. Voltage fluctuation factors are analyzed to establish a fault detection method using pairwise smart meter Diffs, supported by a derived multiple linear equation between Diffs and current. Specifically, the LOF method identifies arc faults by detecting significant outliers in the Diffs, while the MLR method quantifies contact resistance within the metering enclosure’s line segments through the multivariate linear relationship between voltage–current differentials, enabling loose contact diagnosis. Finally, the robustness of the proposed model is validated using collected resistor load and appliance load datasets, and experiments with other data-driven methods, such as LOF alone, MLR alone, *K*-means-MLR, DBSCAN-MLR, IF-PR, and SVM methods, also demonstrate that the proposed LOF-MLR method exhibits strong robustness and diagnostic performance under various datasets. Based on the fault diagnosis results, this study can provide early fault warnings for the safe operation management of smart meters. Future work will explore the application of smart meter data in equipment health management and other areas.

## Figures and Tables

**Table 1:** Different current portfolios.

	Load Type	Normal Contact	Loose Contact	Arc Fault *
M1	Resistors	0 A/0.4 A/3.8 A/7.8 A	0 A/0.4 A/3.8 A/7.8 A	0 A/3.8 A/7.8 A
	Appliances	0 A/(0.15~0.35 A)/(0.25~3.11 A)	0 A/(0.15~0.35 A)/(0.25~3.11 A)	(0.15~0.35 A)/(0.25~3.11 A)/5 A
M2	Resistors	0 A/0.4 A/3.8 A/7.8 A	0 A/0.4 A/3.8 A/7.8 A	0 A/3.8 A/7.8 A
	Appliances	0 A/(0.15~0.35 A)/(0.25~3.11 A)	0 A/(0.15~0.35 A)/(0.25~3.11 A)	(0.15~0.35 A)/(0.25~3.11 A)/5 A
M3	Resistors	0 A/0.4 A/3.8 A/7.8 A/11.6 A	0.4 A/3.8 A/7.8 A/11.6 A	0.4 A/3.8 A
	Appliances	5 A	5 A	(0.15~0.35 A)/(0.25~3.11 A)/5 A

* For safety reasons, the current during this stage is lower than that during other stages.

**Table 2:** The smart meter terminal contact resistance is determined using the MLR method.

Resistance	Normal Contact			Loose Contact			Arc Fault		
	DS1	DS2	DS3 *	DS4	DS5	DS6 *	DS7	DS8	DS9 *
M1 (mΩ)	76.9	76.6	71.1	53.9	88.1	82.1	88.4	55.2	66.4
M2 (mΩ)	30.2	9.8	57.8	31.6	29.9	27.7	26.4	6.9	5.2
M3 (mΩ) *	50.0	49.0	77.3	136.8	270.3	131.2	512.8	431.8	699.4

* The loose contact and arc fault operations are conducted on the M3 terminals. The datasets DS3, DS6, and DS9 are collected under appliance load conditions, while the remaining datasets are obtained under resistor load conditions.

**Table 3:** Comparison with deterministic methods in terminal contact fault diagnosis.

Load Type	Method	*PPV*	*TPR*	*FAR*	*F1*	Diagnostic Error Count
Resistors	LOF	0.98	0.97	0.06	0.98	2
	MLR	0.75	0.89	0.34	0.81	2
	LOF-MLR	0.98	0.97	0.06	0.98	1
	*K*-means-MLR	0.97	0.55	0.06	0.70	4
	DBSCAN-MLR	1	0.90	0	0.95	1
	IF-PR	0.87	1	0.09	0.93	2
	SVM	0.86	1	0.17	0.92	2
Appliances	LOF	0.92	1	0.05	0.96	2
	MLR	0.79	0.85	0.27	0.82	1
	LOF-MLR	0.92	1	0.05	0.96	0
	*K*-means-MLR	1	0.69	0	0.82	2
	DBSCAN-MLR	0.64	1	0.09	0.78	4
	IF-PR	0.88	1	0.20	0.94	1
	SVM	1	0.58	0.23	0.73	2

## Data Availability

The data presented in this study are available on request from the corresponding author.

## Abbreviations

The following abbreviations are used in this manuscript:

AMI	Advanced metering infrastructure
Diffs	Voltage differentials
LOF	Local Outlier Factor
MLR	Multiple Linear Regression
L	Live wire
N	Neutral wire
IF	Isolation Forest
RP	Receptacles
ID	Identification code
*FPG*	Fluctuation in the power grid
*FTC*	Fluctuation caused by changes in $I_{S}$
*FBC*	Variation in branch load currents
*MEs*	Measurement errors of the smart meters
*FAF*	Voltage fluctuation due to arc fault
*FLF*	Voltage fluctuation due to loose contacts
*VF*	Voltage fluctuation
Norm-Arc	Normal contact smart meter and arc fault smart meter
Norm-Norm	Normal contact smart meter and normal contact smart meter
Norm-Loose	Normal contact smart meter and loose contact smart meter
DTW	Dynamic Time Warping
SKF	Skewness features
∆*V*/∆*I*	Voltage-to-current difference ratios
*TP*	True Positive
*FP*	False Positive
*FN*	False Negative
*TN*	True Negative
PR	Polynomial Regression
